# Stereotactic MRI-guided radiation therapy for localized prostate cancer (SMILE): a prospective, multicentric phase-II-trial

**DOI:** 10.1186/s13014-022-02047-w

**Published:** 2022-04-15

**Authors:** J. Ristau, J. Hörner-Rieber, C. Buchele, S. Klüter, C. Jäkel, L. Baumann, N. Andratschke, H. Garcia Schüler, M. Guckenberger, M. Li, M. Niyazi, C. Belka, K. Herfarth, J. Debus, S. A. Koerber

**Affiliations:** 1 Department of Radiation Oncology, Heidelberg University Hospital, Im Neuenheimer Feld 400, 69120 Heidelberg, Germany; 2 Heidelberg Institute of Radiation Oncology (HIRO), Department of Radiation Oncology, Heidelberg University Hospital, Heidelberg, Germany; 3 National Center for Tumor Diseases (NCT), Heidelberg University Hospital, Heidelberg, Germany; 4 Clinical Cooperation Unit Radiation Oncology, German Cancer Research Center (DKFZ), Heidelberg, Germany; 5 Heidelberg Ion-Beam Therapy Center (HIT), Department of Radiation Oncology, Heidelberg University Hospital, Heidelberg, Germany; 6 German Cancer Consortium (DKTK), Core Center, Heidelberg, Germany; 7 Institute of Medical Biometry, Heidelberg University Hospital, Heidelberg, Germany; 8 Department of Radiation Oncology, University Hospital Zurich, University of Zurich, Rämistrasse 100, 8091 Zurich, Switzerland; 9 Department of Radiation Oncology, University Hospital, LMU Munich, Munich, Germany

**Keywords:** Prostate cancer, SBRT, Ultrahypofractionation, MR guided radiotherapy, Adaptive radiotherapy, SIB

## Abstract

**Background:**

Normofractionated radiation regimes for definitive prostate cancer treatment usually extend over 7–8 weeks. Recently, moderate hypofractionation with doses per fraction between 2.2 and 4 Gy has been shown to be safe and feasible with oncologic non-inferiority compared to normofractionation. Radiobiologic considerations lead to the assumption that prostate cancer might benefit in particular from hypofractionation in terms of tumor control and toxicity. First data related to ultrahypofractionation demonstrate that the overall treatment time can be reduced to 5–7 fractions with single doses > 6 Gy safely, even with simultaneous focal boosting of macroscopic tumor(s). With MR-guided linear accelerators (MR-linacs) entering clinical routine, invasive fiducial implantations become unnecessary. The aim of the multicentric SMILE study is to evaluate the use of MRI-guided stereotactic radiotherapy for localized prostate cancer in 5 fractions regarding safety and feasibility.

**Methods:**

The study is designed as a prospective, one-armed, two-stage, multi-center phase-II-trial with 68 patients planned. Low- and intermediate-risk localized prostate cancer patients will be eligible for the study as well as early high-risk patients (cT3a and/or Gleason Score ≤ 8 and/or PSA ≤ 20 ng/ml) according to d’Amico. All patients will receive definitive MRI-guided stereotactic radiation therapy with a total dose of 37.5 Gy in 5 fractions (single dose 7.5 Gy) on alternating days. A focal simultaneous integrated boost to MRI-defined tumor(s) up to 40 Gy can optionally be applied. The primary composite endpoint includes the assessment of urogenital or gastrointestinal toxicity ≥ grade 2 or treatment-related discontinuation of therapy. The use of MRI-guided radiotherapy enables online plan adaptation and intrafractional gating to ensure optimal target volume coverage and protection of organs at risk.

**Discussion:**

With moderate hypofractionation being the standard in definitive radiation therapy for localized prostate cancer at many institutions, ultrahypofractionation could be the next step towards reducing treatment time without compromising oncologic outcomes and toxicities. MRI-guided radiotherapy could qualify as an advantageous tool as no invasive procedures have to precede in therapeutic workflows. Furthermore, MRI guidance combined with gating and plan adaptation might be essential in order to increase treatment effectivity and reduce toxicity at the same time.

## Background

In 2021, the estimated number of new prostate cancer cases was 248,530 in the US accounting for 26% of all new cancer cases in males [1]. Worldwide, the number of new prostate cancer cases was 1,414,259 in 2020 [2]. Due to its high prevalence and demographic changes towards growing proportions of elderly people in many western countries, there is a need for effective treatment alternatives, both in terms of outcome and economic burden of the health care system. Also, the COVID-19 pandemic has shown that reduced treatment times can be beneficial with regard to limited treatment capacities. In primary treatment of prostate cancer, definitive radiation therapy plays a major role. Apart from surgical options, various treatment options are available for intermediate-risk (IR) and low-risk (LR) disease, including active surveillance and brachytherapy for the latter. Normofractionated image-guided intensity-modulated radiotherapy (IG-IMRT) has been the standard radiation scheme at many institutions, usually extending over 7–8 weeks [3]. Moderate hypofractionation using single doses between 2.4 and 3.4 Gy/fraction has been shown to achieve very good clinical outcomes compared to normofractionated radiotherapy in patients with prostate cancer [4–7]. The CHHiP trial, one of the largest of these studies, having included more than 3000 patients with localized prostate cancer, demonstrated noninferiority of moderate hypofractionation with 60 Gy in 20 fractions compared to conventional fractionation [4]. Therefore, moderate hypofractionation has become a recommended alternative in the German S3-guideline. Radiobiologic considerations based on the linear-quadratic model lead to the assumption that prostate cancer might in particular benefit from hypofractionation in terms of tumor control and toxicity. The α/β value as measure of fractionation sensitivity is considered to be relatively low for prostate cancer cells in relation to its surrounding normal tissues, predicting higher sensitivity to fractional doses without compromising toxicity. Several preclinical and clinical studies have suggested α/β values of about 1.5 Gy for prostate cancer cells, which would theoretically lead to a better therapeutic ratio [8–13]. Considering the overall treatment time as another factor in calculation of the α/β value results in higher values of about 2–2.7 Gy [14]. In recent years, many patients have been treated with ultrahypofractionated radiation regimes, reducing treatment to 3–10 fractions with a major part of published data focusing on regimes with only 5 fractions. Those studies showed excellent biochemical control rates of 93–100% after 1–5 years in patients with LR or IR disease [15–22]. Widmark et al. were the first to publish 5-year-results of a randomized phase III study, demonstrating non-inferiority of ultrahypofractionated radiation with 42.7 Gy in seven fractions 3 days per week compared to 78 Gy in 39 fractions with regard to failure-free survival and late toxicity [23]. When using normofractionation, the addition of a focal boost up to 95 Gy to MR-defined macroscopic tumor(s) improved biochemical disease-free survival (bDFS) in the recent FLAME phase III trial without compromising toxicity in patients with IR and HR disease [24]. Furthermore, first toxicity data from the phase II hypo-FLAME trial showed that the addition of simultaneous focal boosting to the macroscopic tumor(s) to ultrahypofractionated SBRT of the prostate gland is safe with no grade ≥ 3 acute GU or GI toxicity and acceptable rates of grade 2 toxicities[25].

As MR-guided linear accelerators (MR-linacs) have entered clinical routine, new aspects in terms of image guidance and plan adaptation promise to further improve stereotactic body radiation therapy (SBRT) outcomes. Whereas cone-beam CTs or other X-ray based systems involve radiation exposure, image guidance using integrated MR imaging does not. One of the major advantages using magnetic resonance-guided radiotherapy (MRgRT) is the ability to visualize target volumes such as the prostate or organs at risk (OAR) before, after but also during radiation therapy [26]. This seems to increase accuracy of targeting and enables sparing of radiation dose to OAR in particular when using hypofractionation. First published data of clinical use are promising. In locally advanced pancreatic cancer, SBRT with 24 Gy in 3 fractions could safely be applied without higher grade toxicity [27, 28]. Data by Rudra et al. found that high dose SBRT (biologically effective dose [BED_10Gy_] > 70 Gy) using adaptive MRgRT for treatment of inoperable pancreatic cancer patient resulted in improved overall survival rates compared to standard doses [29]. We see great potential in the use of MRgRT for prostate SBRT, as OAR such as the rectum can be monitored closely in order to reduce therapy-related toxicity. On the other hand, the target volume itself can be displayed with excellent quality, which could result in higher treatment effectivity [30].

The aim of this prospective, multicentric phase II trial is to evaluate the incidence and grade of genitourinary (GU) and gastrointestinal (GI) toxicity and the incidence of treatment-related discontinuations of therapy in patients with local LR and IR as well as early high-risk prostate cancer patients. It is the first prospective phase II trial combining MR-guided SBRT with optional simultaneous focal boosting of macroscopic tumor(s). The greater aim is to generate data for following trials, which could in the long term establish MRgRT in combination with ultrahypofractionation as a potential new time-saving and economically favorable option for the named patient groups.

## Methods

### Study design

The study is designed as a prospective, one-armed, two-stage, multi-center phase-II-trial, evaluating the safety and feasibility of extreme hypofractionated radiotherapy with MRgRT in localized prostate cancer. After obtaining written informed consent, patient fulfilling the inclusion criteria will undergo treatment planning with non-contrast enhanced simulation MR imaging at the linac and standard computer tomography. Multi-parametric magnetic resonance imaging (mpMRI) of the prostate is included in the study protocol as an optional procedure. Fiducials should not be implanted into the prostate gland. Radiation therapy is applied as MRI-guided intensity-modulated radiation therapy (IMRT) with gated dose delivery. Online plan adaptation should be performed at every treatment session. Systemic treatments, e.g. androgen deprivation therapies are not part of this study and should be handled according to current therapy guidelines.

### Study objectives

The hypothesis of our study is that stereotactic MRI-guided radiotherapy is safe for patients with low-, intermediate- and early high-risk prostate cancer. The primary endpoint of our study is a composite endpoint with the occurrence of either any genitourinary (GU) or gastrointestinal (GI) toxicity ≥ grade 2 within one year after start of radiotherapy or treatment-related discontinuation of therapy being an event. Secondary endpoints include treatment-related mortality within one year and within 5 years after start of radiation therapy, GU and GI toxicity graded according to NCI CTCAE version 5.0 after one and five years from treatment start, biochemical progression free survival (bPFS) since treatment start defined as PSA recurrence according to the phoenix criteria (post-therapeutic nadir of PSA + 2 ng/ml) and androgen deprivation therapy (ADT) free survival (3 months of neoadjuvant ADT is allowed). Furthermore, secondary endpoints include overall survival (OS) and quality of life (QOL), assessed with EORTC QLQ-C30 und -PR25 questionnaires during and after treatment. Planning and treatment parameters as well as imaging and quality assurance results are assessed for further evaluation.

### Patient population/patient selection

Inclusion criteria according to the protocol are:

- Histologically confirmed prostate cancer with Gleason-score grading and PSA parameters
- Low-/intermediate- and early high-grade (cT3a/and or Gleason Score 8 and/or PSA ≤ 20 ng/ml) risk group according to d’Amico criteria
- International Prostate Symptom Score (IPSS) of ≤ 12
- Prostate gland volume < 80 cm^3^
- Karnofsky Index ≥ 70%
- Age ≥ 18 years
- Written informed consent

Exclusion criteria are the following:

- Patient’s refusal or incapability of informed consent
- Prior pelvic radiation therapy
- Prior local therapy of the prostate gland
- Lymphogenic metastases
- Stage IV (distant metastases)
- Contraindication to undergo MRI
- Participation in another clinical trial which might influence the results of the SMILE study

### Sample size calculation

In order to estimate the sample size of our prospective phase II trial, we followed data published by Bruynzeel et al. [30]. In their MRgRT prostate SBRT trial with 5 × 7.25 Gy, the maximum cumulative grade ≥ 2 early GU and GI toxicity was 23.8%. We hypothesize that the rate of treatment-related therapy discontinuations can be neglected. It is our goal to show that the rate of events is below a clinically acceptable limit of 40%. A sample size of 68 patients with a planned interim analysis after 30 patients was calculated in order to be able to reject the null hypothesis at the one-sided significance level of α = 0.025 with a statistical power of 80%. We chose Simon’s two stage design to reduce the expected number of patients under the null hypothesis compared to a single stage design, as a premature study termination with acceptance of the null hypothesis is possible [31, 32]. If the null hypothesis is true, the expected sample size is 42. As a discontinuation of therapy is included in the primary composite endpoint, missing values are not to be expected. The sample size calculations were performed using R (Version 3.6.1) with the R package OneArmPhaseTwoStudy [33].

### Statistical analysis

The statistical analysis includes all enrolled patients (Intention-to-treat- (ITT) population). Additionally, a per-protocol- (PP) analysis will be performed. Since this is an exploratory study all results will be interpreted descriptively. An interim analysis will be conducted when 30 patients are evaluable for the primary endpoint. If an event is observed in at least 11 of these patients (corresponding to 37%), the study will be terminated with acceptance of the null hypothesis. In case of 10 or fewer events the study will be continued with recruitment of 38 additional patients.

A 95% confidence interval, correcting for the two-stage design, will be calculated for the event rate of the primary endpoint. Kaplan–Meier curves will be computed for all survival endpoints. Regression models will be used to analyze factors influencing the different endpoints. Missing values will not be imputed.

### Contouring and dose specifications, MRgRT delivery

**Fig. 1:**
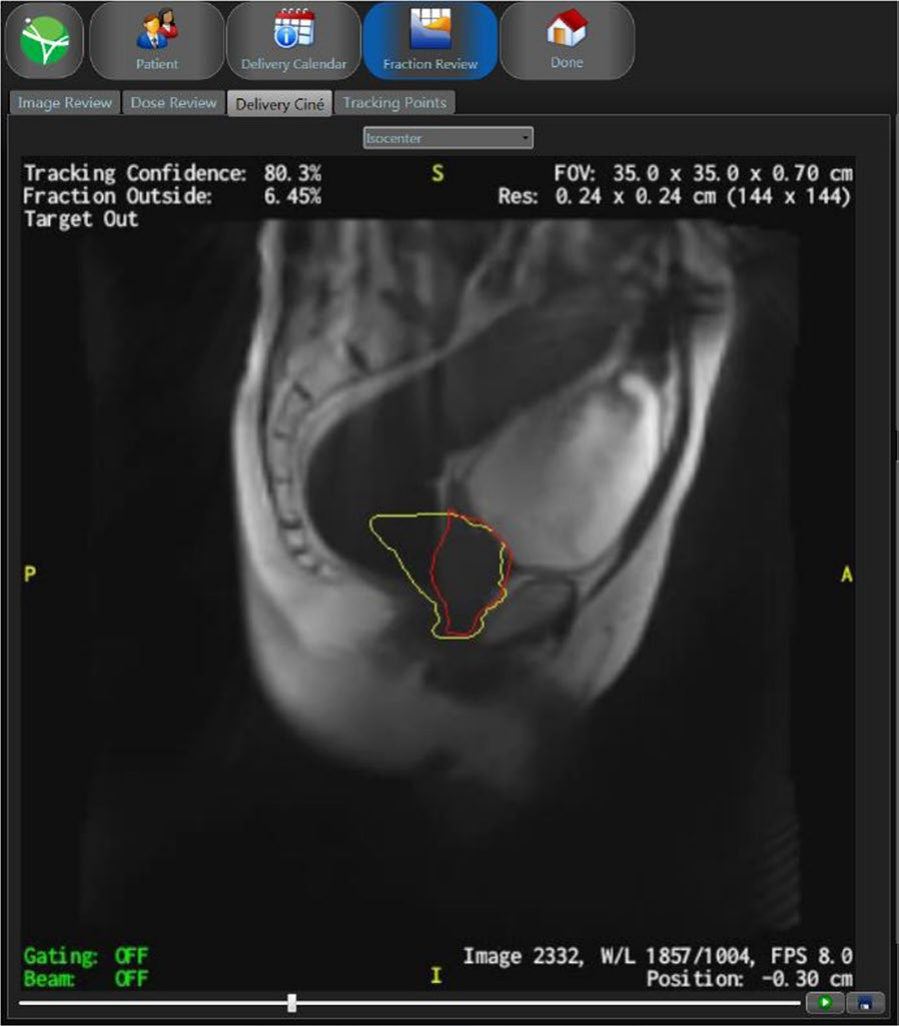
Cine MRI sequence. Yellow structure: Gating margin (CTV + 3 mm). Red structure: deformably registered gating target. This screenshot is showing the instance when a large amount of rectal air is moving the prostate out of the gating margin, automatically shutting off beam delivery

All patients are treated at a 0.35 Tesla (T) MRIdian Linac® system (ViewRay Inc., Oakwood, USA) with a 6-megavolt linear accelerator [34]. To prepare for the first MR planning scan, patients are instructed to have their bladder about half full (in house standard protocol: 250 ml of water 30 min before scan). A high-resolution MRI scan at the MRIdian linac with 1.5 mm × 1.5 mm × 1.5 mm resolution with the patient in supine position is then acquired using a True FISP sequence with T2/T1-weighted contrast. For patient positioning, suitable immobilization devices such as knee cushions and footrests are used to generate reproduceable positioning. Delineation of the target volume and organs at risk is based on this planning scan. For dose calculation purposes, all patients undergo a computed tomography planning scan using the same positioning tools immediately after the MR simulation (usually within one hour). The simulation MRI scan is co-registered with the mpMRI sequences (T2w, DWI and DCE) in treatment position. The CTV includes the prostate and in case of intermediate risk profile the base of the seminal glands. A 3 mm margin is added to create the PTV. In case of optional simultaneous focal boost to macroscopic tumor(s), the GTV contains the dominant intraprostatic lesion(s) based on mpMRI according to the Prostate Imaging – Reporting and Data System Version 2 (PI-RADS™ v2) [35]. A PTV margin of 0–2 mm is added to the SIB GTV (at investigator discretion). Contouring of the GTV will be performed in collaboration with experienced uroradiologists. Apart from the usual pelvic organs at risk, the posterior third of the rectal wall should explicitly be delineated. Goal of the treatment plan is to achieve the best possible coverage of the target volume with maximal protection of the organs at risk. Radiotherapy is delivered every other day with a minimum time in between of 36 h. The total treatment time should not exceed 14 days. A homogenous dose of 37.5 Gy in 5 fractions (dose per fraction: 7.5 Gy) is prescribed to the PTV, corresponding to an EQD_2_^(α/β: 2)^ of 89.0 Gy. RT plans are prescribed to the median dose aiming at 95% coverage of the PTV by 95% of the prescribed target dose. A dose per fraction of 8.0 Gy is prescribed to macroscopic tumor(s), in case of optional focal boosting. Maintaining OAR dose constraints is of first priority, accepting decreased GTV coverage and/or dose in case of predicted violation of constraints. Organ at risk constraints are based on data that have previously been published for prostate ultra-hypofractionation (Table 1) [30, 36]. MRI-based image guidance, Cine MR-enabled anatomy tracking and beam gating is performed at every treatment session according to the center’s standard operation procedures followed by online adaptation with re-contouring of organs at risks and target volume at every fraction (Fig. 1). The radiation plan should be adapted at every session. OAR contours and pre-treatment CT imaging are deformably registered to the MRI of the day using a vendor-supplied algorithm. CTV contours are then manually adapted by the attending radiation oncologist, as well as OAR contours in a region expanding 1 cm in cranio-caudal direction and 3 cm in all other directions from the PTV (PTV_expand_) on the MRI of the day [37].

**Table 1:** Dose constraints

OAR	Constraint
Urethra + 2 mm	D_0.2 cc_ ≤ 37.5 Gy
Bladder	D_0.2 cc_ ≤ 38.5 Gy
	D_mean_ ≤ 25 Gy
Rectum	D_0.2 cc_ ≤ 38.5 Gy
Bowel	D_0.5 cc_ ≤ 35 Gy

### Follow-Up

Participants are assessed according to the current German S3 guidelines for at least 5 years. Measurement of PSA is scheduled after study enrollment (baseline), 4–6 weeks after start of therapy, every 3 months during the first 2 years after treatment and twice a year during years 3–5. PSA measurements should always be carried out by the same laboratory in order to avoid variation due to differing measurement methods. Regular study follow-up visits at the treating institution include (Table 2):

- update of medical history
- assessment of symptoms and treatment-related toxicity according to NCI CTCAE version 5.0
- assessment of quality of life using EORTC QLQ-C30 and QLQ-PR25 questionnaires
- optional multiparametric MR imaging 6 weeks, 6 months and 12 months after start of treatment

**Table 2:** follow up scheme

Timepoint	Study period																			
	Pre-treatment		Study treatment		Follow-up															
	Enrolment	RT planning	RT start (wk 1)	RT end (wk 2)	wk 6	wk 12	6 mths after RT	9 mths after RT	12 mths after RT	15 mths after RT	18 mths after RT	21 mths after RT	24 mths after RT	30 mths after RT	36 mths after RT	42 mths after RT	48 mths after RT	54 mths after RT	60 mths after RT	In case of recurrence
Eligibility screen	x																			
Informed consent	x																			
Medical history	x		x	x	x	x	x	x	x		x		x		x				x	x
Symptoms	x		x		x	x	x	x	x		x		x		x				x	x
PSA level*	x				x	x	x	x	x	x	x	x	x	x	x	x	x	x	x	x
MR Linac simulation	x																			
Toxicity			x	x	x	x	x	x	x		x		x		x				x	x
CT pelvis		x																		
MRI pelvis**		x																		
EORTC QoL questionnaires	x			x	x	x	x	x	x		x		x		x				x	x
Blood test	x																			

*PSA can be tested at treating urologist or at study center (according to local standard operating procedures)

**Optional

### Trial organization and coordination

The SMILE study has been designed by the study initiators at the department of Radiation Oncology in cooperation with the Institute of Medical Biometry at the Heidelberg University Hospital. The department of Radiation Oncology is in charge of the overall trial management, database management, quality assurance including monitoring and reporting. The study investigators are experienced radiation oncologists specialized in the treatment of patients with urogenital malignancies. Patients will be recruited and treated by the physicians of the departments of Radiation Oncology at the University Hospital Heidelberg, the University Hospital Munich (LMU) and the University Hospital Zurich (Switzerland).

### Ethics, informed consent and safety

The final protocol was approved by the ethics committee of the University of Heidelberg, Heidelberg, Germany (Nr.: S-915/2020; Munich: 21-0662; Zurich: 2021-D0032). The SMILE study complies with the Helsinki Declaration in its recent German version, the principles of Good Clinical Practice (GCP) and the General Data Protection Regulation (GDPR) as well as the Federal Data Protection Act (FDPA). The trial will also be carried out in accordance with local legal and regulatory requirements. The ClinicalTrials.gov identifier is NCT04845503.

## Discussion

The Scandinavian trial by Widmark et al. is the only study so far that has reported non-inferior outcome and toxicity 5 years after normofractionated vs. ultrahypofractionated radiotherapy for intermediate-risk and a small proportion of high-risk prostate cancer patients [23]. Another non-inferiority trial, PACE-B, compared conventionally fractionated, moderately hypofractionated radiotherapy (78 Gy in 39 fractions over 7–8 weeks or 62 Gy in 20 fractions over 4 weeks, respectively) and SBRT with 36.25 Gy in five fractions over 1–2 weeks. For PACE-B early toxicity results are available, showing no significant difference between the treatment arms with a slight trend in favor of the SBRT regime (23% vs. 27%) [38]. In the HYPO-RT-PC trial, acute grade 2 or worse genitourinary toxicity was slightly but not significantly higher in the SBRT (42.7 Gy in 7 fractions) arm (28% vs. 23%, p = 0.057) compared to normofractionation (78 Gy in 39 fractions) at the end of radiation therapy. The difference was significant after one year (6% vs. 2%, p = 0.0037), but disappeared after five years. GI toxicity was not significantly different at any timepoint. MR imaging for target volume delineation or usage of IMRT were not mandatory in that trial, resulting in a large proportion of patients treated with 3D-conformal RT [23]. In PACE-B, Cyberknife and conventional LINACs were used with mandatory IGRT and intra-fractional motion control. The only prospective data for MRgRT with an ultrahypofractionated treatment regime were published by Bruynzeel et al., reporting relatively low early GI and GU toxicity (23.8% and 5.0% respectively for the maximum cumulative grade ≥ 2 toxicity at any study time point), with the majority of enrolled patients having high-risk disease (59.4%) [30]. Bruynzeel et al. reported a peak of GU toxicity at the end of treatment and discussed that their chosen 6 weeks follow up time point may have obscured very early increases in toxicity after the end of RT. Therefore, we planned a first assessment of toxicity 4 weeks after the end of RT (Table 1) [39]. The FLAME phase III trial has demonstrated that focal boosting to intraprostatic lesion in normofractionated RT is safe and improves bDFS (92% vs. 85% after 5 years) without compromising toxicity and quality of life compared to standard treatment without boost [24]. The same group’s hypo-FLAME trial is now following up patients treated with SBRT (35 Gy in 5 weekly fractions) with an integrated boost up to 50 Gy to mpMRI-defined tumor lesions. First published toxicity data are promising, showing no acute ≥ grade 3 toxicities and acceptable 34.0% and 5.0% grade 2 GU and GI toxicities [25]. MRgRT generally offers various advantages such as using small CTV to PTV margins, online CTV monitoring, daily plan reoptimization and the avoidance of gold marker implantation. The latter is in particular beneficial for patients, obviation additional invasive procedures with encompassing risks such as infection. On the other hand, it is more time-consuming and cost intensive compared to other SBRT techniques. Questions regarding cost-effectivity will have to be answered in future analyses. The aim of our study is to assess the safety and feasibility of ultrahypofractionated MRgRT in a prospective setting. The innovative approach of our trial clearly is the combination of MRgRT with ultrahypofractionated SBRT, allowing focal integrated boosting of mpMRI-defined tumor lesions.

## Data Availability

Not applicable.
